# Use of Contrast-Enhanced Ultrasound in Suspected Traumatic or Spontaneous Renal Injury in Cats: A Case Series

**DOI:** 10.3390/ani15213089

**Published:** 2025-10-24

**Authors:** Simone Perfetti, Carolina Gai, Nikolina Linta, Giacomo Tamburini, Erika Monari, Elena Ciuffoli, Alessia Diana

**Affiliations:** 1 Department of Veterinary Medical Sciences, University of Bologna, Via Tolara di Sopra 50, 40064 Ozzano dell’Emilia, Italy; simone.perfetti4@unibo.it (S.P.); nikolina.linta2@unibo.it (N.L.); elena.ciuffoli3@unibo.it (E.C.); 2 AniCura Istituto Veterinario di Novara, 28060 Granozzo con Monticello, Italy; gai.carolina@live.it; 3 Independent Researcher, 40100 Bologna, Italy; giacomotamburini1@gmail.com

**Keywords:** cats, contrast-enhanced ultrasound, renal trauma, veterinary imaging

## Abstract

**Simple Summary:**

Traumatic injuries caused by penetrating or blunt trauma account for approximately 10% of mortality in companion animals. In pediatric human patients, the kidney is the third most frequently injured abdominal organ during trauma. In human medicine, contrast-enhanced ultrasound (CEUS) is a well-established technique for assessing traumatic injuries involving parenchymal organs. While CEUS is increasingly utilized in veterinary medicine, its application in renal assessment remains underexplored, particularly in the context of trauma. This study describes CEUS findings in three cases of traumatic or spontaneous renal rupture in cats. Two cases presented ultrasonographic and CEUS features consistent with renal hematomas or lacerations, while one case showed active subcapsular renal hemorrhage. Hematomas appeared as focal disruptions of renal architecture, often with cystic, non-enhancing areas on CEUS. Active bleeding was characterized by contrast extravasation in subcapsular or retroperitoneal locations. Overall, CEUS appeared to be a rapid, safe, and minimally invasive method for evaluating traumatic and/or spontaneous renal injuries and may represent a useful complementary tool in the diagnostic approach to feline renal trauma. However, the findings of this study should be interpreted cautiously given the small number of cases.

**Abstract:**

Contrast-enhanced ultrasound (CEUS) is increasingly applied in veterinary medicine as a safe, rapid, and non-invasive imaging technique for assessing renal disorders. Despite its expanding use, the literature on its application in feline renal trauma remains scarce. This retrospective study aimed to describe CEUS findings in cats with suspected traumatic renal injuries. Medical records were reviewed for cats that underwent both B-mode ultrasonography and CEUS, with findings confirmed by follow-up, surgery, or cytology. Three cats met the inclusion criteria. Two presented focal or multifocal renal lesions ranging from 10 to 20 mm in diameter, with heterogeneous echotexture, distortion of renal contours, and non-enhancing areas on CEUS consistent with hematomas or lacerations. The third cat showed a circumferential subcapsular halo (approximately 3–5 mm thick) with evidence of contrast leakage, compatible with limited active hemorrhage. CEUS appeared effective in identifying and characterizing renal injuries, offering valuable information to support clinical decision-making and guide both conservative and surgical management. Nevertheless, due to the limited sample size and the absence of quantitative data, these results should be considered preliminary. Further prospective studies are warranted to confirm the diagnostic performance and clinical utility of CEUS in feline renal trauma.

## 1. Introduction

Traumatic injuries resulting from blunt or penetrating abdominal trauma are an important cause of acute mortality in both companion animals and humans, with reported mortality rates of up to 10% in animals [1,2,3,4]. The kidneys, because of their retroperitoneal position and rich vascular supply, are particularly vulnerable to trauma. In children, they are the third most commonly affected abdominal organ after the spleen and liver, during blunt abdominal trauma [2,3]. In human medicine, contrast-enhanced computed tomography (CT) is considered the gold standard for detecting trauma-related injuries [3]. However, its use in veterinary patients is limited by high costs and, most importantly, the requirement for general anesthesia. This condition represents a significant risk in unstable or critically injured animals, making CT less feasible in emergency settings. Consequently, abdominal focused assessment with sonography for trauma (AFAST) has become a widely used first-line diagnostic tool in small animals, particularly for the detection of abdominal effusion in polytraumatized patients [5]. Nevertheless, the absence of abdominal effusion does not rule out trauma, and AFAST may miss solid organ injuries such as concussive or contusive lesions in the absence of active bleeding [5,6]. In this context, contrast-enhanced ultrasonography (CEUS) provides a safe, radiation-free, and cost-effective alternative capable of identifying parenchymal organ injuries, including ruptures, hematomas, lacerations, and active hemorrhage [7]. Its diagnostic role in human blunt abdominal trauma—particularly renal trauma—is well established [6,7,8,9]. In veterinary medicine, CEUS has gained increasing relevance for evaluating parenchymal perfusion in physiological and pathological renal conditions, offering high temporal resolution and real-time assessment of microvascular flow. Several studies in dogs and cats have demonstrated its value in the evaluation of acute kidney injury, chronic kidney disease, and renal perfusion abnormalities [10,11,12,13,14]. However, only a few reports have explored its use in traumatic or spontaneous renal injury, especially in feline patients [15]. Therefore, the aim of this study was to describe CEUS findings in suspected cases of renal trauma/rupture in cats and to assess its value as a reliable tool for the early diagnosis of renal injuries, and to provide preliminary observational data that may support future prospective studies on small animal renal trauma.

## 2. Materials and Methods

Medical records of cats admitted to the Veterinary Teaching Hospital of the University of Bologna between 2019 and 2024 were retrospectively reviewed. Animals were included if they had a suspected or confirmed history of traumatic events and/or imaging findings consistent with trauma-related renal lesions. Inclusion criteria required that each patient had undergone a two-dimensional (B-mode) abdominal ultrasound with specific attention to the urinary system, followed—either concurrently or subsequently—by contrast-enhanced ultrasound (CEUS) of the kidneys. For all included cases, data were collected on signalment, clinical history, physical examination findings, laboratory results, and follow-up data. To meet inclusion criteria, B-mode and CEUS findings suggestive of renal trauma had to be supported by at least one of the following: surgical confirmation, cytological and/or histopathological evaluation (exclusion of other underlying disease, i.e., neoplastic), or ultrasonographic follow-up demonstrating progressive resolution or improvement of the renal abnormalities. B-mode and CEUS findings suggestive of renal trauma were considered focal or multifocal disruption of the normal renal architecture with or without an expansile lesion, characterized by heterogeneous echotexture possibly containing cavitary, fluid-filled structures, and lacking contrast enhancement on CEUS. Any extravasation of contrast medium from the renal parenchyma was considered consistent with active bleeding. Cats were included if CEUS was performed within 72 h of presentation for suspected renal trauma or spontaneous rupture. Exclusion criteria were the presence of pre-existing renal neoplasia or congenital malformations affecting kidney morphology. The timing of CEUS relative to trauma was recorded whenever information from owners or referring veterinarians was available.

B-mode and CEUS evaluations were performed using an ultrasound system (EPIQ5G, Philips Healthcare, Monza, Italy) equipped with convex (2–9 MHz), micro-convex (5–8 MHz), and linear (5–12 MHz) transducers. When available, Color Doppler findings were also evaluated. Ultrasonographic images were reviewed offline by two of the authors (SP & AD). CEUS examinations were conducted using high-frequency linear-array (L5–12 MHz) or convex (2–9 MHz) probes, following a previously described protocol (14-15). The same contrast medium (sulfur hexafluoride; SonoVue^®^, Bracco, Milano, Italy) was used for each exam. The contrast medium was administered manually through an indwelling cephalic venous 22G catheter as a rapid bolus of 0.5 mL, followed immediately by a rapid bolus of 4 mL saline. The kidney was imaged in longitudinal and transverse planes, with cine loops recorded for at least 90 s to capture the entire wash-in and wash-out phases. All scans were stored in DICOM format for subsequent analysis. Image and cine loop reviews were performed using a dedicated Picture Archiving and Communication System (PACS) (Carestream Vue PACS, Carestream Health, Rochester, NY, USA). All ultrasonographic examinations, including both B-mode and CEUS, were qualitatively evaluated by experienced operators in accordance with current veterinary ultrasound and CEUS recommendations. Each renal lesion identified on B-mode ultrasound was evaluated for shape, margins, echotexture and echogenicity. Assessment also included the integrity of the corticomedullary architecture in the affected renal portion, the appearance of the remaining renal parenchyma, and any associated alterations in the perirenal retroperitoneal space. CEUS analysis was based on a qualitative assessment, evaluating the presence or absence of contrast enhancement within the lesion, the homogeneity or heterogeneity of enhancement, and any evidence of contrast medium extravasation into subcapsular or perirenal retroperitoneal spaces.

Hematology and biochemistry assays were performed using automated analyzers (hematology: ADVIA 2120, Siemens Healthcare Diagnostics, Tarrytown, NY, USA; biochemistry: OLYMPUS AU480, Olympus/Beckman Coulter, Brea, CA, USA). Coagulation profiles were determined using an automated coagulometer (BCS XP, Siemens Healthcare Diagnostics, Tarrytown, NY, USA) with species-specific reagents and controls. Reference intervals for all parameters were those validated internally by the diagnostic laboratory of the University of Bologna.

Descriptive statistics were applied to summarize demographic, clinical, and clinicopathological data. Continuous variables are expressed as median and range. Due to the small sample size, no further statistical analysis was performed. A summary of the main variables is provided in Table 1.

The study was conducted retrospectively on client-owned cats presented for clinical evaluation. Ethical review and approval were waived because all diagnostic procedures were performed as part of routine clinical care. Written informed consent for imaging procedures and the use of anonymized clinical data for research was obtained from all owners.

## 3. Results

A review of the medical records identified three cats who underwent B-mode and CEUS for suspected renal trauma or spontaneous renal rupture.

A structured summary of the main clinical and clinicopathological features of the three cases is reported in Table 1.

### 3.1. Case 1

**Figure 1:**
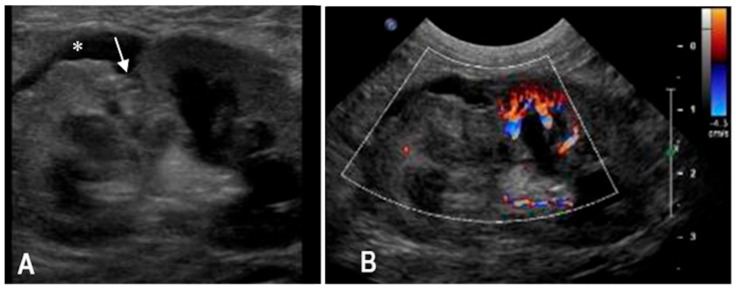
(**A**) B-mode and (**B**) Color Doppler ultrasonography of the left kidney in a cat with traumatic renal laceration (case 1). (**A**) Architectural disruption of the cranial pole with cortical depression (white arrow), surrounded by a subcapsular anechoic area (*), is evident. (**B**) Color Doppler of the same region showed absence of vascular signals in the cranial pole, consistent with non-perfused parenchyma.

**Figure 2:**
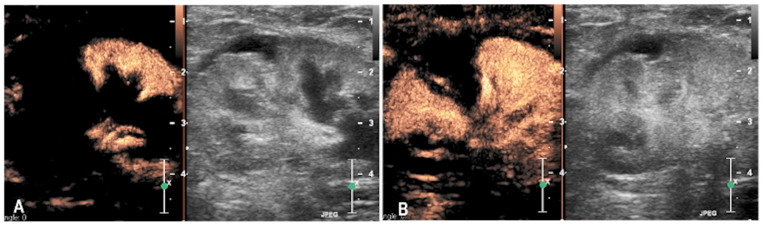
Representative contrast-enhanced ultrasound (CEUS) sequences of the left kidney in a cat with traumatic renal laceration (case 1). Each sequence illustrates the contrast-enhanced image on the left and the corresponding grayscale image on the right. (**A**) During the cortical phase, the cortex of the caudal pole shows homogeneous enhancement, while the cranial pole remains avascular. (**B**) In the corticomedullary phase, homogeneous enhancement of the renal parenchyma is observed in the caudal pole and in part of the cranial pole. A triangular area between the two poles remains non-enhancing, consistent with non-perfused tissue.

A 5-year-old intact female domestic shorthair cat was presented approximately one hour after falling from a second-floor window. The referring veterinarian had noted the presence of pigmented urine. Upon admission, the cat exhibited mildly labored and shallow respiration (respiratory rate: 60 breaths per minute) and pale mucous membranes. Thoracic auscultation revealed slightly diminished breath sounds bilaterally, while abdominal palpation elicited pain in the left renal region. An abrasion was also noted on the left hind limb. The remainder of the physical examination was unremarkable. Complete blood count highlighted severe anemia (HCT 17%, RI 32–48%) and mild leukopenia (WBC 4470 × 10^3^/μL, RI 4.8–14.93 × 10^3^/μL). Serum biochemistry revealed mildly increased creatinine (2.62 mg/dL, RI 0.80–1.80 mg/dL) and urea (127 mg/dL, RI 30–65 mg/dL) concentrations, together with an increase of liver and muscle parameters (ALT 490 U/L, RI 20–72 U/L); AST 607 U/L, RI 9–40 U/L) and a decrease of albumin concentration (Albumin 1.81 g/dL; RI 2.6–4 g/dL). Urinalysis demonstrated the presence of red blood cells in the sediment. Thoracic radiographs identified a moderate bilateral pneumothorax, more pronounced on the left side. Abdominal radiographs revealed an apparent enlargement of the left kidney, irregular renal margins, and subtle increased radiopacity of the perirenal fat. Retrograde urethrocystography excluded lower urinary tract rupture. Abdominal ultrasonography confirmed enlargement of the left kidney with architectural disruption of the cranial pole and associated cortical depression. A subcapsular, irregular, triangular, anechoic area measuring 13 × 10 mm was observed in the cranial renal region, with no detectable blood flow on Doppler imaging. Mild perirenal, retroperitoneal and peritoneal effusions were also present (Figure 1). CEUS was performed to assess renal perfusion. CEUS demonstrated normal enhancement in the caudal pole of the kidney, while the cranial pole appeared completely avascular. No evidence of contrast medium extravasation was observed in the retroperitoneal space (Figure 2). The lack of enhancement within the cranial pole with sharply demarcated non-perfused areas was consistent with focal parenchymal disruption. Based on the B-mode and CEUS findings, the lesion was interpreted as a traumatic renal laceration with no signs of active bleeding. No cytological samples were performed. A follow-up ultrasound performed four days later showed mild improvement, with resolution of peritoneal and retroperitoneal effusion. However, the cranial pole lesion remained largely unchanged. The cat was discharged on the day of the follow-up examination. No additional clinical or imaging follow-up information was available.

### 3.2. Case 2

**Figure 3:**
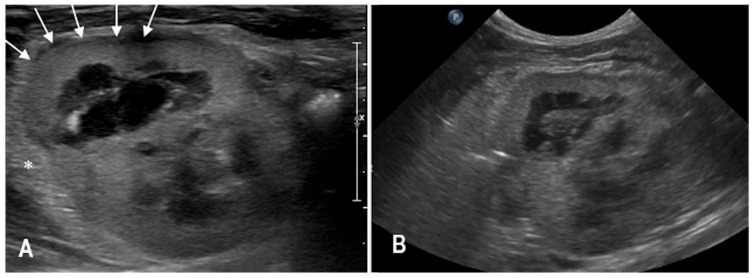
B-mode ultrasonography of the right kidney in a cat with traumatic diaphragmatic hernia (case 2), obtained with a linear probe (**A**) and a microconvex probe (**B**). The kidney shows a severely altered shape; the cranial pole (white arrows) appears globose with heterogeneous echotexture and loss of normal corticomedullary architecture. A thin hypoechoic halo surrounds the entire organ. Diffuse thickening and heterogeneity of the retroperitoneal adipose tissue are also evident (*).

**Figure 4:**
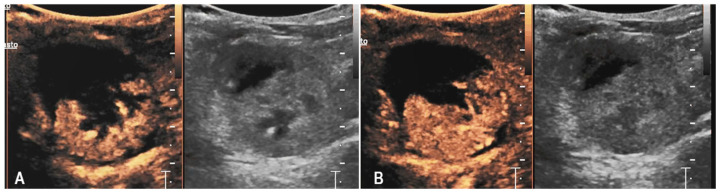
Representative contrast-enhanced ultrasound (CEUS) sequences of the right kidney in a cat with traumatic diaphragmatic hernia (case 2). Each sequence illustrates the contrast-enhanced image on the left and the corresponding grayscale image on the right. (**A**) In the cortical phase, enhancement is mainly observed in the cortex of the caudal pole, while the cranial pole remains largely non-enhancing, consistent with non-perfused parenchyma. (**B**) In the corticomedullary phase, slight heterogeneous contrast enhancement of the renal parenchyma is observed in the caudal pole, whereas the cranial region remains avascular.

A 3-year-old spayed female domestic shorthair cat was presented for evaluation due to lethargy that developed approximately 24 h after returning home. The owner reported a bruise on the right flank and pigmented urine, described as pink-colored urine. On physical examination, the cat exhibited paradoxical breathing and muffled heart sounds, predominantly on the right side. Multiple bruises were also noted. Hematological analysis revealed moderate anemia (HCT 21%, RI 32–48%), while serum biochemistry showed increased urea levels (90 mg/dL, RI 30–65 mg/dL) and marked elevations in hepatomuscular enzymes (ALT 591 U/L, RI 20–72 U/L; AST 418 U/L, RI 9–40 U/L). Urinalysis demonstrated proteinuria (100 mg/dL), glycosuria (>300 mg/dL), and the presence of leukocytes (+) and red blood cells (250/μL) in the sediment. Total-body radiographs revealed a diaphragmatic hernia with suspected intrathoracic herniation of the liver and stomach. Surgical repair was undertaken based on these findings. Intraoperative exploration confirmed the presence of liver, stomach, spleen, and jejunal loops within the thoracic cavity. In addition, a right renal abnormal location was observed, with the right kidney positioned within the peritoneal cavity instead of the retroperitoneal space. The right kidney displayed several traumatic changes, including superficial lacerations along the caudal margin and a deeper lesion at the cranial pole. No active hemorrhage or urinary leakage was detected. A bilateral, extensive retroperitoneal hematoma was also present. Postoperative abdominal ultrasonography revealed a deep indentation along the dorsal margin of the right kidney, with marked cortical-medullary disruption and regional hypoechogenicity. The cranial pole appeared enlarged, with heterogeneous echotexture and loss of corticomedullary definition. Diffuse thickening of the retroperitoneal adipose tissue was also noted (Figure 3). CEUS showed partial parenchymal enhancement of the right kidney, primarily limited to the caudal pole, while the cranial pole remained non-enhancing. Tortuous interlobar arteries were visible during the wash-in phase (Figure 4). Serial follow-up imaging with B-mode and CEUS over the days following surgery showed no significant changes in the renal findings. The patient was discharged three days postoperatively in good clinical condition. At the most recent follow-up, eight days after the initial ultrasound, improved corticomedullary definition was observed in the caudal pole, suggesting partial reperfusion and reorganization of the previously injured area.

### 3.3. Case 3

**Figure 5:**
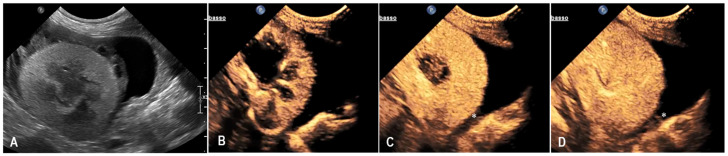
B-mode (**A**) and contrast-enhanced ultrasound (CEUS) sequences (**B**–**D**) of the left kidney in a cat with spontaneous subcapsular hemorrhage (case 3). (**A**) B-mode shows a heterogeneously hypo-anechoic band circumferentially surrounding the left kidney. The kidney displays mildly irregular margins and the cortex is diffusely hyperechoic. (**B**) During the early cortical phase, the renal cortex enhances homogeneously, while the subcapsular band remains avascular. In the early (**C**) and late (**D**) corticomedullary phases, the renal parenchyma enhances homogeneously. The subcapsular band persists as a well-demarcated, non-enhancing structure, while a mild subcapsular contrast leak (*) is visible, consistent with limited active bleeding.

A 10-year-old neutered male Chartreux cat, previously managed at the clinic for Addison’s disease and hypertrophic cardiomyopathy, was presented for the sudden onset of disorientation and lethargy. On clinical examination, the cat showed altered mentation with pronounced disorientation. An emergency blood test revealed severe anaemia, with a packed cell volume (PCV) of 11%, leading to an immediate transfusion of red blood cells. Further diagnostics included hematobiochemical testing and an abdominal ultrasound. The biochemical profile highlighted increases in creatinine (3.42 mg/dL; reference range 0.80–1.80 mg/dL), urea (124 mg/dL; reference range 30–65 mg/dL), ALT (87 U/L; reference range 20–72 U/L), and AST (114 U/L; reference range 9–40 U/L). Coagulation times were also altered, with a prothrombin time (PT) of 15.8 s (RI 9–15 s) and an activated partial thromboplastin time (aPTT) of 25.3 s (RI 9–20 s), both above the normal ranges. Abdominal ultrasonography revealed a diffusely increased echogenicity of the left kidney, with a circumferential, irregular, and heterogeneously hypoechoic subcapsular halo that showed no vascular signal on Color Doppler evaluation. Additionally, the perirenal adipose tissue appeared heterogeneously echogenic, and a mild retroperitoneal effusion was observed on the right side. The right kidney presented only a diffuse increase in parenchymal echogenicity. Based on the clinical background and diagnostic findings, the ultrasound features were considered primarily compatible with renal hemorrhage, although acute renal inflammation could not be definitively excluded. The patient remained hospitalized and received supportive therapy tailored to the various comorbidities. During the hospital stay, the cat’s clinical condition fluctuated. A follow-up ultrasound performed one week later showed progression of the previously described left renal subcapsular component, with a notable increase in the fluid-like portion. Because of the suspicion of subcapsular hemorrhage, CEUS was performed. CEUS showed homogeneous enhancement of the renal parenchyma while the subcapsular halo remained persistently avascular throughout all phases, with a faint focal contrast leakage suggesting mild ongoing bleeding (Figure 5). Given the limited extent of hemorrhage seen on CEUS, a conservative management approach was chosen. The patient’s condition stabilized during hospitalization, and he was discharged twelve days after the CEUS. Unfortunately, a few days later, the cat died spontaneously.

## 4. Discussion

This case series describes the combined B-mode and CEUS features observed in three cats with suspected renal laceration or rupture, either traumatic or spontaneous in origin, and discusses how the two sonographic modalities can be integrated at the bedside to help triage and management. A history of confirmed or strongly suspected blunt trauma was present in two cases, likely secondary to high-rise falls (one case confirmed by the owner) and/or a car accident. The remaining case had no known history of trauma at the time of presentation; however, retrospective analysis of clinical history and progression supported either traumatic or spontaneous rupture (due to parenchymal damage) as the likely etiology. Across all three cases, B-mode ultrasonography identified focal or multifocal alterations of renal architecture characterized by distortion of the normal renal contour and heterogeneous echotexture with intra-lesional hypo-/anechoic components likely representing hemorrhagic or necrotic areas. In some instances, these lesions mimicked expansile masses. Retroperitoneal involvement, either in the form of fluid accumulation or organized tissue suggestive of hematoma, was also observed, compatible with hemorrhagic infiltration or edema, or contusion of the retroperitoneal, peri-renal adipose tissue. These findings framed the suspicion of parenchymal damage, localized the compartment (intraparenchymal vs. subcapsular vs. perirenal/retroperitoneal), and helped us plan targeted CEUS acquisitions over the suspicious area. On CEUS, all suspected lesions contained non-enhancing intraparenchymal or subcapsular areas consistent with non-perfused tissue, likely due to hematoma, laceration, or necrosis. These features align with previous descriptions in pediatric human patients, in which renal parenchymal injuries appear as sharply marginated, avascular regions on CEUS [7,8,9]. The absence of enhancement within the affected renal regions reliably distinguished viable parenchyma from non-perfused areas, while the presence or absence of contrast leakage was a key criterion for differentiating static hematoma from active bleeding [8,9]. In small animal medicine, CEUS has been increasingly used to assess renal perfusion under both physiological and pathological conditions. In healthy dogs and cats, CEUS shows a typical renal perfusion pattern with rapid cortical enhancement and gradual medullary filling, providing a reference for recognizing true perfusion deficits [10]. In dogs with acute kidney injury, reduced cortical enhancement and slower wash-in indicate impaired blood flow [9], while in cats with chronic kidney disease, delayed and diminished cortical perfusion reflects altered cortical–medullary hemodynamics [11]. Although CEUS has been widely explored in physiological and non-traumatic renal disorders, its use in renal trauma remains only marginally investigated in small animals [12]. Most available data derive from experimental canine studies or isolated case reports, and feline evidence is quite lacking. When applied to acute renal injury, CEUS has proven particularly valuable in differentiating viable from non-perfused tissue and detecting active bleeding. In an experimental canine model of hemorrhagic shock, Lin et al. [14] showed that CEUS accurately depicted renal lacerations as sharply demarcated non-enhancing regions corresponding to hematoma or necrosis. Case reports in dogs with spontaneous or traumatic renal rupture described similar findings—avascular intra-parenchymal or subcapsular regions and, in some instances, localized contrast extravasation confined to the subcapsular or retroperitoneal compartment [13,15]. In our series, the CEUS appearance of feline renal trauma mirrored these previously described canine patterns. In all cats, CEUS revealed distinct non-enhancing regions corresponding to parenchymal or subcapsular hematomas. In one cat, a faint subcapsular contrast leak was observed, indicating a contained active bleeding, whereas in the remaining cats, no contrast extravasation was detected despite marked morphologic disruption on B-mode. The ability of CEUS to differentiate perfused from non-perfused parenchyma and to localize bleeding within specific anatomical compartments (subcapsular versus perirenal or retroperitoneal) proved clinically relevant for decision-making and follow-up. Two of the cats in this series presented traumatic lacerations that were managed conservatively with supportive care and follow-up ultrasonography. In both, CEUS confirmed the absence of active bleeding, thereby avoiding unnecessary surgical intervention. In the third case, CEUS confirmed a suspicion of renal subcapsular active bleeding due to renal spontaneous rupture secondary to coagulation alterations. CEUS identified a mild focal contrast leak, confirming limited active bleeding and supporting also in this case conservative management. No adverse reactions to the contrast agent were observed. The agent’s favorable safety profile—being non-nephrotoxic and non-hepatotoxic—makes it particularly suitable for use in patients with suspected renal dysfunction or hemodynamic instability [16]. Unlike iodinated contrast agents used in computed tomography (CT), which are excreted renally and may pose a risk in cases of impaired renal function, CEUS provides a safer alternative in hemodynamically unstable patients. Some limitations of this study should be considered when interpreting the results. The small number of included cases does not allow for statistical analysis and inevitably restricts the possibility of drawing broad conclusions; however, it reflects the rarity of documented feline renal trauma cases investigated with CEUS in clinical practice. The retrospective nature of the work limited the degree of standardization achievable in-patient selection, image acquisition, and follow-up intervals. Nonetheless, all examinations were performed using a standardized CEUS protocol, and data were reviewed by experienced operators to reduce potential variability. Another limitation concerns the qualitative assessment of CEUS findings. Quantitative perfusion analysis could have provided additional information, but the authors consider a qualitative assessment sufficient in the emergency clinical context, where rapid information regarding renal parenchymal perfusion and potential contrast extravasation is crucial for clinical decision-making. Finally, advanced imaging (i.e., contrast-enhanced CT) or histopathology was not available for all patients, which prevented full confirmation of the imaging diagnoses. Despite these constraints, the present study offers preliminary results on the diagnostic utility and clinical applicability of CEUS for evaluating suspected renal trauma in cats and may serve as a basis for future prospective research. Overall, the current evidence from experimental, clinical, and chronic disease studies supports CEUS as a versatile and minimally invasive imaging tool capable of providing both structural and functional information about renal integrity. Within this context, our feline cases add novel clinical data demonstrating that CEUS can be applied successfully to identify and monitor renal lacerations in cats, even in the absence of advanced imaging modalities such as CT. Its non-nephrotoxic nature, bedside feasibility, and ability to monitor perfusion changes over time make CEUS a particularly attractive option in feline patients with traumatic or concurrent chronic renal disease.

## 5. Conclusions

In conclusion, CEUS appeared to be a safe, rapid, and minimally invasive imaging technique for evaluating suspected traumatic or spontaneous renal injuries in cats. The combination of B-mode ultrasonography and CEUS allowed the identification of renal lesions such as lacerations, hematomas, or subcapsular hemorrhages, and provided real-time assessment of parenchymal perfusion. Lacerations and hematomas appeared as expansile, non-enhancing areas that disrupted normal renal architecture. CEUS may serve as a valuable diagnostic tool in the initial evaluation of trauma patients, guiding therapeutic decisions and helping to avoid unnecessary surgical intervention. Although these results are based on a limited number of cases, they support the potential role of CEUS as an adjunct imaging modality in feline renal trauma, especially when CT is contraindicated or unavailable. Future prospective studies with larger cohorts and quantitative analysis are encouraged to further validate these preliminary findings and to refine CEUS diagnostic criteria for renal injury in small animals.

## Figures and Tables

**Table 1:** Summary of demographic, clinical, and clinicopathological findings in three feline cases with traumatic or spontaneous renal injury. Reference intervals (RI) are reported in parentheses. Median and range values for key clinicopathological parameters are presented in the summary row.

Case	Age/Sex/Breed	History/Trauma	Main Clinical Findings	Clinical Pathology Findings (RI)
1	5 y/F/Domestic shorthair	Fall from 2nd floor	Tachypnea pale mucosae pain left kidney palpation	HCT 17% (32–48) WBC 4.47 × 10^3^/μL (4.8–14.93) Creatine 2.62 mg/dL (0.8–1.8) Urea 127 mg/dL (30–65) ALT 490 U/L (20–72) AST 607 U/L (9–40) Alb 1.81 g/dL (2.6–4.0) RBCs in urine sediment
2	3 y/F spayed/Domestic shorthair	Suspected blunt trauma Diaphragmatic rupture	Lethargy paradoxical breathing pink urine	HCT 21% (32–48) Urea 90 mg/dL (30–65) ALT 591 U/L (20–72) AST 418 U/L (9–40) RBCs in urine sediment
3	10 y/M neutered/Chartreux	No trauma Addison’s disease HCM	Lethargy	PCV 11% (32–48) Creat 3.42 mg/dL (0.8–1.8) Urea 124 mg/dL (30–65) ALT 87 U/L (20–72) AST 114 U/L (9–40) PT 15.8 s (9–15) aPTT 25.3 s (9–20)
Summary (median/range)	Age: 5 y (3–10) Sex: 2F/1M	Lesion types: 2 traumatic 1 spontaneous;		HCT/PCV: 17% (11–21) Creatinine: 3.02 mg/dL (2.62–3.42) Urea: 124 mg/dL (90–127) ALT: 490 U/L (87–591) AST: 418 U/L (114–607)

F, female; M, male; Alb, albumin; ALT, alanine aminotransferase; AST, aspartate aminotransferase; aPTT, activated partial thromboplastin time; HCT, hematocrit; PCV, packed cell volume; PT, prothrombin time; RBCs, red blood cells; RI, reference interval; WBC, white blood cells.

## Data Availability

The data presented in this study are available on request from the corresponding author. The data are not publicly available due to privacy restrictions related to client-owned animals.

## Abbreviations

The following abbreviations are used in this manuscript:

AFAST	Abdominal Focused Assessment with Sonography for Trauma
ALT	Alanine aminotransferase
AST	Aspartate aminotransferase
aPTT	Activated partial thromboplastin time
B-mode	Brightness mode ultrasonography
CEUS	Contrast-enhanced ultrasonography
CT	Computed tomography
HCM	Hypertrophic cardiomyopathy
PCV	Packed cell volume
PT	Prothrombin time
RI	Reference interval
RBCs	Red blood cells
